# The resistance‐compliance relationship at low pulmonary resistance: Integrating pulmonary wedge pressure into right ventricular load assessment

**DOI:** 10.14814/phy2.70811

**Published:** 2026-03-08

**Authors:** Hannah Kempton, Nick Olsen, Katherine Kearney, Christopher S. Hayward, David W. M. Muller, Audrey Adji

**Affiliations:** ^1^ Faculty of Health and Medicine The University of New South Wales Sydney New South Wales Australia; ^2^ Department of Cardiology St Vincent’s Hospital Sydney New South Wales Australia; ^3^ Department of Cardiology University of Texas Southwestern Medical Center Dallas Texas USA; ^4^ Victor Chang Cardiac Research Centre Sydney New South Wales Australia

**Keywords:** pulmonary arterial compliance, pulmonary hypertension, pulmonary vascular resistance

## Abstract

The inverse hyperbolic relationship between pulmonary vascular resistance (PVR) and pulmonary arterial compliance (PAC) is a well‐established marker of right ventricular (RV) afterload. Pulmonary capillary wedge pressure (PCWP) is a known modifier of this relationship; however, its impact across pulmonary hypertension (PH) subgroups has not been well characterized. How does PCWP influence the resistance‐compliance (RC) relationship in PH subgroups, and can it be integrated into PAC calculations? Data from routine right heart catheterizations were analyzed to evaluate the PVR‐PAC relationship across PH subgroups, both with and without PCWP adjustment. The inverse PVR‐PAC relationship was apparent in cohorts with low PVR (*p* < 0.001). In isolated post‐capillary PH (Ipc‐PH), this relationship was PCWP‐dependent (unadjusted: *p* = 0.084; adjusted: *p* = 0.008). Incorporating PCWP significantly improved model fit (*R* ^2^ from 0.725 to 0.919). The RC relationship is broadly preserved across PH but is modulated by PCWP in low PVR states, particularly Ipc‐PH. Adjusting PAC calculations for PCWP may enhance the clinical assessment of RV afterload using this metric.

## 1 INTRODUCTION

In contrast to the left ventricle, the right ventricle (RV) is a relatively low‐pressure chamber, which is highly sensitive to the afterload from the pulmonary circulation. While afterload is routinely estimated clinically by the mean pulmonary arterial pressure (mPAP) and pulmonary vascular resistance (PVR), the pulsatility or compliance of the pulmonary arterial system also makes a significant contribution to RV work and afterload. Numerous methods for measuring this pulsatile load have been proposed. One of the most frequently described is pulmonary arterial compliance (PAC). PAC can be calculated using the empiric formula as the stroke volume (SV) divided by pulmonary pulse pressure (PP) and has been related to the severity and prognosis of pulmonary vascular disease (Pellegrini et al., 2014; Tampakakis et al., 2018).

The PAC and PVR therefore estimate the pulsatile and resistive components of RV afterload respectively, and an inverse hyperbolic resistance‐compliance (RC) relationship between the two parameters has been well described (Lankhaar et al., 2006, 2008; Lim & Gustafsson, 2020; MacKenzie Ross et al., 2013; Naeije & Delcroix, 2014; Tedford, 2014; Tedford et al., 2012). The RC relationship is such that at low or normal PVR, PAC makes a greater contribution to the global RV load than at higher PVR, where the higher resistive load plays a more dominant role (Hungerford et al., 2024; Tedford, 2014; Wang et al., 2023). This may explain why PAC is a stronger predictor of outcome than PVR in heart failure cohorts, where the PVR is typically lower than, for example, in primary pulmonary arterial hypertension cohorts (Al‐Naamani et al., 2015; Pellegrini et al., 2014).

The RC relationship is also modified by the left atrial pressure, measured as the pulmonary capillary wedge pressure (PCWP). As the PCWP rises, the transluminal pressure in the pulmonary vessels increases, reducing their compliance (Tedford et al., 2012). Mechanistically, the rising PCWP increases pulmonary arterial pressure, distending the pulmonary vessels, and reducing the PAC (Naeije & D'Alto, 2016). However, at lower PVR, where the pulmonary vessels remain highly compliant, the rising PCWP is not transmitted directly to the pulmonary arteries, but rather buffered by distension of the pulmonary vascular bed (Naeije & D'Alto, 2016). The PCWP is therefore not directly reflected in the pulmonary pulse pressure. The effect of PCWP on PAC can be considered in terms of the effect of the PCWP on the pulse pressure. Considering that PAC = SV/PP, in patients with PCWP that is elevated in excess of the diastolic pulmonary arterial pressure (DPAP), the PAC will be overestimated. Recognizing this, this study describes the effect of PCWP on PAC in pulmonary hypertension (PH) sub‐groups, characterizing the effect of PCWP on the RC relationship in these subgroups. It hypothesizes that the effect will be most significant in the post‐capillary PH cohort, where the PCWP is most significantly elevated, and explores the effect of incorporating PCWP into the calculation for PAC.

## 2 METHODS

Data from patients who underwent clinically indicated right heart catheterization between 2021 and 2024 was retrospectively analyzed. The study was prospectively reviewed and approved by the institutional ethics committee, and written informed consent was obtained from all patients. To reflect real‐world data, cases were excluded only if hemodynamic values were unfeasible, reflecting recording error. Patients were classified into PH groups, according to guideline definitions, with the exception of the inclusion of those patients with mean pulmonary arterial pressure (mPAP) 20–25 mmHg included with the normotensive group (Marc et al., 2022). Pulmonary pressures were measured using a fluid filled catheter, and included the systolic pulmonary arterial pressure (SPAP), diastolic pulmonary arterial pressure (DPAP), mean pulmonary arterial pressure (mPAP), and mean PCWP. The pulmonary arterial pulse pressure (PP) was calculated as SPAP − DPAP, the transpulmonary gradient (TPG) as mPAP − PCWP, and the diastolic pressure gradient (DPG) as DPAP – PCWP. Cardiac output (CO) was calculated using the thermodilution method, with measurements repeated until three values were obtained within 10% of one another, and an average taken. Where there was significant variability, or severe tricuspid regurgitation, the estimated Fick method was used. The SV was calculated as the CO divided by heart rate. PAC was calculated using the formula (SV/PP), and the PVR as TPG divided by CO.

According to the aims of this study, the empiric PAC formula was modified to incorporate PCWP into the denominator (Equation 1), thereby accounting for downstream pressure in the calculation of pulse pressure.

Alternative approaches to incorporating PCWP into the PAC calculation were also explored, including substitution of the DPG and setting DPG to zero when negative. Given that the primary objective was to account explicitly for PCWP, the PCWP‐based formulation was retained for the main analyses. Results of these sensitivity analyses are provided in the (Figure S1).

(1) $${\mathrm{PAC}}_{\text{pcwp}}=\frac{\mathrm{SV}}{\mathrm{PP}+\text{PCWP}}$$

where SV is the stroke volume and PP is the pulse pressure (the difference between the systolic and diastolic pulmonary arterial pressures), and the PCWP is the pulmonary capillary wedge pressure.

Subgroups analyzed in this study included: (i) normal pulmonary pressures, or those with borderline elevated pulmonary pressures (20–25 mmHg) and normal PCWP and PVR; (ii) post‐capillary PH (Ipc‐PH); (iii) pre‐capillary PH; (iv) combined pre‐ and post‐capillary PH (Cpc‐PH); and (v) patients with PVR <3.0 WU, where the PAC could be expected to be intact due to the relatively low resistive load.

### 2.1 Statistical analysis

Statistical analysis was conducted in R version 4.4.1. A series of generalized linear mixed models with gamma distribution and inverse link functions were applied to evaluate the RC relationship across the entire cohort, and then in subsets of PH groups including those with normal or borderline PH, lower PVR (<3WU), isolated post‐capillary PH (Ipc‐PH), combined pre‐ and post‐capillary PH (Cpc‐PH), and pre‐capillary PH, to characterize the RC relationship in these subgroups. Simple regression was performed between PAC and PVR. Multiple regression was used to adjust for PCWP, as well as age and body surface area (BSA), due to their known effect on pulmonary hemodynamics. The significance of PCWP as a modifier of the PVR‐PAC (RC) relationship was evaluated using model performance metrics and likelihood ratio tests including an Akaike information criterion (AIC) and Bayesian information criterion (BIC), with lower numbers indicating better model fit, as well as ANOVA and pseudo‐*R* ^2^ statistics where applicable. Modeling was also applied to determine the impact of PAC_PCWP_ as compared to PAC on the RC relationship, including in PH sub‐groups.

## 3 RESULTS

**FIGURE 1:**
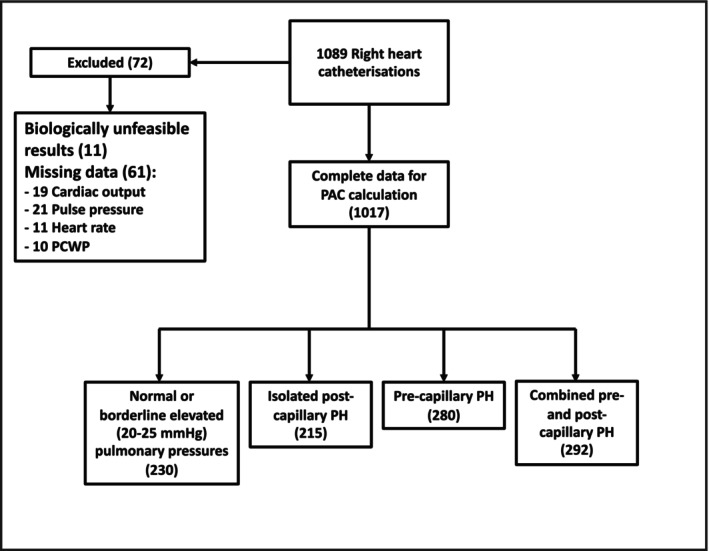
Study exclusions.

Results from 1089 right heart catheterizations (RHC) were obtained for all patients undergoing clinically indicated RHC at a single center. 72 cases were excluded due to incomplete or physiologically unfeasible data (19 CO; 21 PP, 11 heart rate, 10 PCWP, and 11 biologically unfeasible values, for example mPAP 1001), leaving 1017 cases with complete data (Figure 1). Baseline demographic and hemodynamic data for the cohort is presented in Table 1. The average age was 61.6 ± 16.4, 470 patients (46%) were female. The cohort included 230 (23%) with normal pulmonary hemodynamics, 215 (21%) with isolated post‐capillary PH, 280 (28%) with pre‐capillary PH, and 292 (29%) with combined pre‐ and post‐capillary PH. The mean PAC was 2.98 ± 1.90 mL/mmHg, and mean PVR 3.4 ± 3.0 WU.

**TABLE 1:** Baseline characteristics.

*N* = 1017	Characteristic (mean ± SD)
Age (years)	61.6 ± 16.4
Gender (female), *n* (%)	470 (46%)
Pulmonary hypertension classification, *n* (%)	
Pre‐capillary	280 (28%)
Isolated post‐capillary	215 (21%)
Combined pre‐and post‐capillary	292 (29%)
Normal or borderline* PH	230 (23%)
BMI (kg/m^2^)	28.1 ± 6.7
BSA (m^2^)	1.90 ± 0.26
Heart rate (bpm)	75 ± 15
SAP (mmHg)	126 ± 23
DAP (mmHg)	71 ± 12
MAP (mmHg)	89 ± 14
SPAP (mmHg)	51 ± 21
DPAP (mmHg)	23 ± 10
mPAP (mmHg)	32 ± 13
RAP (mmHg)	12 ± 6
PCWP (mmHg)	18 ± 8
TPG (mmHg)	14 ± 11
CO (L/min)	4.7 ± 1.5
CI (L min m^2^)	2.5 ± 0.8
SV (mL)	66 ± 23
PVR (dynes)	268 ± 242
PVR (WU)	3.4 ± 3.0
PAC_l_ (mL/mmHg)	2.98 ± 1.90
SVR (WU)	17.8 ± 6.4

Abbreviations: BMI, body mass index; BSA, body surface area; CI, cardiac index; CO, cardiac output; DAP, diastolic arterial pressure; DPAP, diastolic pulmonary arterial pressure; MAP, mean arterial pressure; mPAP, mean pulmonary arterial pressure; PCWP, pulmonary capillary wedge pressure; PVR, pulmonary vascular resistance; RAP, right atrial pressure; SAP, systemic arterial pressure; SPAP, systolic pulmonary arterial pressure; SV, stroke volume; SVR, systemic vascular resistance; TPG, transpulmonary gradient.

* (mPAP 20–25, PCWP <15 mmHg; PVR <2.0).

Results for the regression analyses are displayed in Table 2, and model comparison statistics are in Table S1, for each of the pre‐defined sub‐cohorts.

**TABLE 2:** Results of PVR versus PAC regression analyses.

Model	Intercept (β_0_)	PAC coefficient ($\beta_{1}$)	95% CI	Z score	*p*‐value	Sample size
Entire cohort (*n* = 1017)						
1	0.53	2.18	2.02; 2.34	27.42	<0.0001	1017
2*	−1.33	2.33	2.20; 2.46	35.64	<0.0001	1017
Normal/borderline PH (*n* = 230)						
3	6.43	1.29	0.90; 1.68	6.48	<0.0001	230
4*	−0.44	1.39	0.95; 1.83	6.17	<0.0001	230
Ipc‐PH (*n* = 215)						
5	11.02	0.45	−0.06; 0.97	1.73	0.084	215
6*	10.13	0.83	0.22; 1.45	2.65	0.008	215
Low PVR cohort; PVR <3.0 WU (*n* = 624)						
7	6.34	1.07	0.56; 1.13	9.11	<0.0001	624
8*	5.90	1.42	1.13; 1.70	9.68	<0.0001	624
Pre‐capillary PH (*n* = 280)						
9	−0.15	1.56	1.44; 1.68	25.89	<0.0001	280
10*	−0.22	1.56	1.41; 1.71	20.50	<0.0001	280
Cpc‐PH (*n* = 292)						
11	2.7	1.05	0.82; 1.29	8.91	<0.0001	292
12*	0.01	1.24	0.95; 1.52	8.48	<0.0001	292

Abbreviations: Cpc‐PH, combined pre‐ and post‐capillary pulmonary hypertension; Ipc‐PH, Isolated post‐capillary pulmonary hypertension; PAC, pulmonary arterial compliance; PH, pulmonary hypertension; PVR, pulmonary vascular resistance; WU, wood units.

* Multiple‐regression, adjusted for PCWP, body surface area, and age.

**FIGURE 2:**
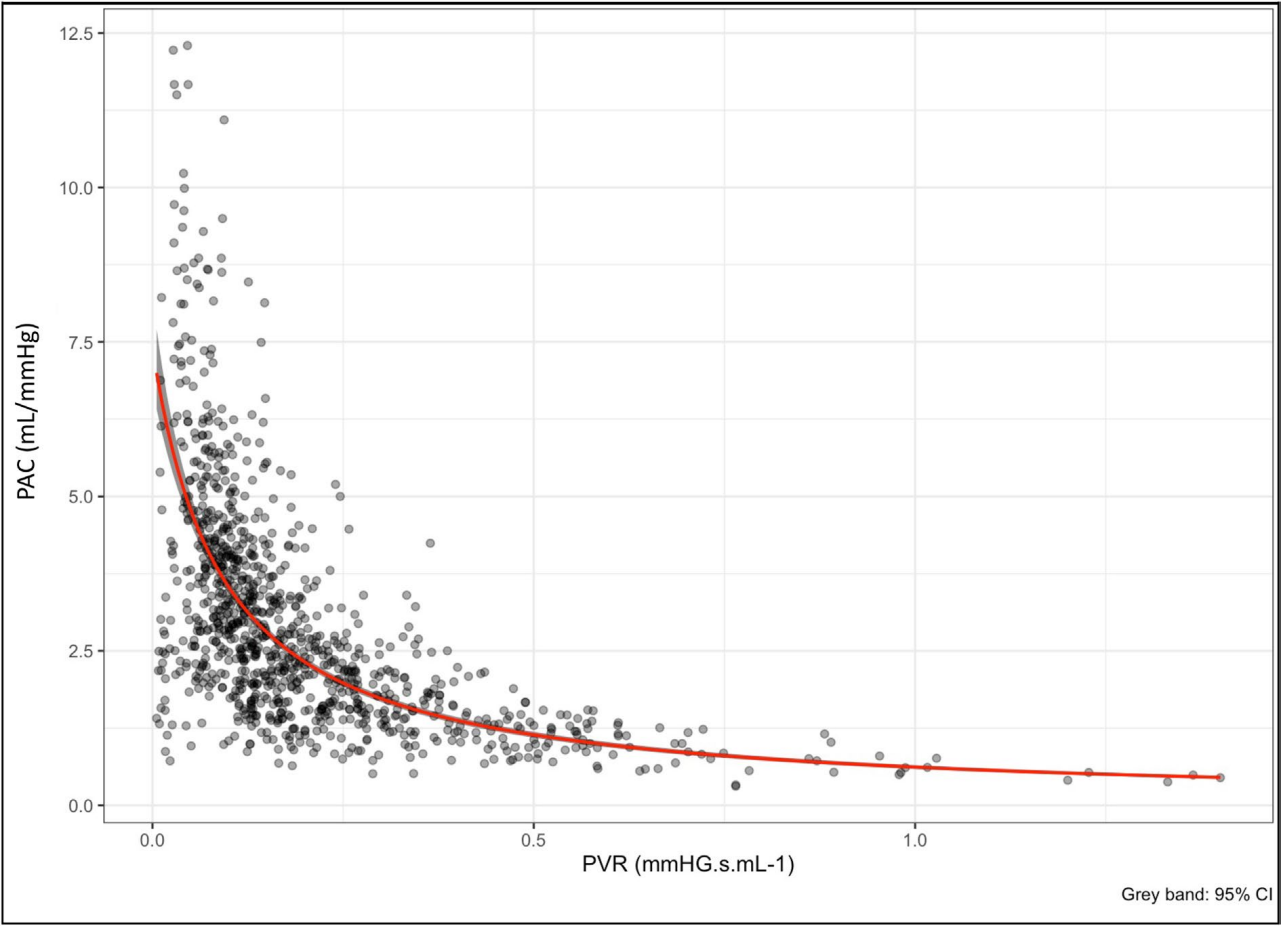
The PAC to PVR (RC) relationship. Gray bands represent confidence intervals.

**FIGURE 3:**
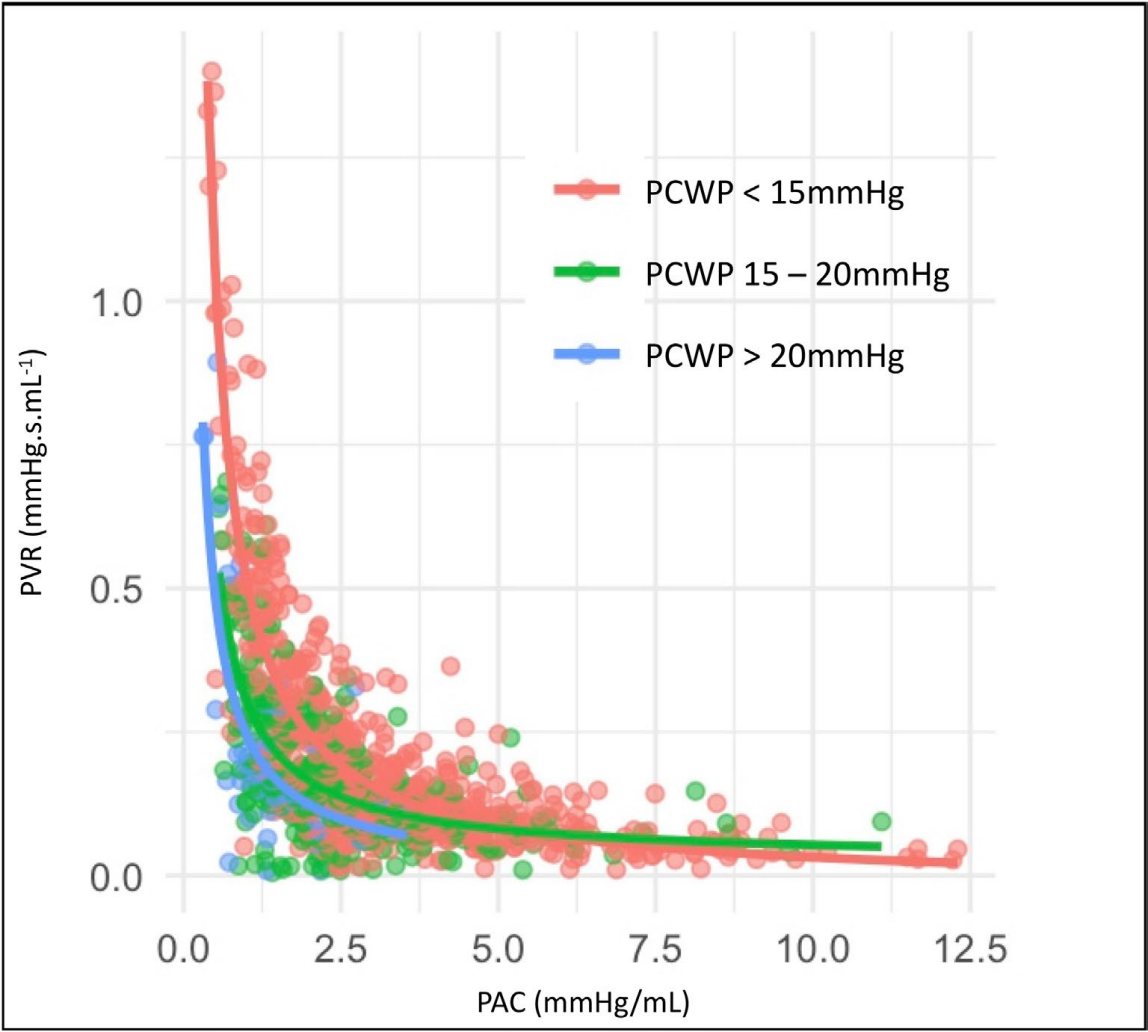
Pulmonary arterial compliance and resistance, stratified by pulmonary capillary wedge pressure. PAC, pulmonary arterial compliance; PCWP, pulmonary capillary wedge pressure; PVR, pulmonary vascular resistance.

In line with findings from previous studies, there was a significant inverse hyperbolic relationship between PAC and PVR ($\beta_{1}$ = 2.18; 95% CI 2.03–2.34, *z* = 27.42, *p* < 0.0001; $\mathrm{PVR}=\frac{1}{0.53+2.18\cdot \mathrm{PAC}}$; Figure 2). Simple regression analyses showed a significant association between PCWP and PAC (*z* = 11.55; *p* < 0.0001), but not PVR (*z* = 0.25; *p* = 0.803) (Tables S2 and S3). Multiple regression analysis showed a persistent significant association between PAC and PVR ($\beta_{1}$ = 2.33; 95% CI 2.20–2.46, *z* = 35.64, *p* < 0.0001), with a significant improvement in model fit and performance with the addition of PCWP (*R* ^2^ = 0.968; *R* ^2^ = 0.985; χ^2^ = 152.71; *p* < 0.0001) (Table 2, Table S1). The relationship between PAC and PVR, stratified by PCWP is shown in Figure 3.

In simple regression analyses, there was a significant inverse hyperbolic relationship between PAC and PVR in each sub‐group, except for the Ipc‐PH cohort ($\beta_{1}$ = 0.45; 95% CI −0.06 to 0.97; *z* = 1.73; *p* = 0.084; Table 2). However, PCWP was significantly associated with PAC in analyses in this subgroup (*z* = 6.35, *p* < 0.001; Table S2), and multiple regression analysis showed a significant inverse hyperbolic relationship between PAC and PVR when adjusted for PCWP ($\beta_{1}$ = 0.83; 95% CI 0.22–1.45, *z* = 2.65, *p* = 0.008; Table 2). There was a significant improvement in the fit and predictive power of the model after adjusting for PCWP (*R* ^2^ = 0.725 to 0.881; χ^2^ = 34.81; df = 1; *p* = 0.028), and the PCWP was a significant modifier of the RC relationship (*z* = 2.61; *p* = 0.031).

In the cohort with normal pulmonary pressures (*n* = 230), PCWP was not significantly associated with PAC (*z* = 0.24; *p* = 0.814), however the multi‐regression analysis produced significantly stronger model fit ($\beta_{1}$ = 1.32, 95% CI 0.95–1.7, *z* = 6.96, *p* < 0.0001; *χ* ^2^ = 27.28, df = 1, *p* < 0.0001; Table 2, Table S1), and again PCWP but not age nor BSA was a significant co‐variate in this model (*z* = 5.37; *p* < 0.0001) (Tables S4–S6).

PCWP was also significantly associated with PAC in subgroups with PVR <3 WU, as well as the Cpc‐PH cohort (Table S2). Regression analysis in the cohort with PVR <3 WU showed a significant inverse hyperbolic relationship between PVR and PAC, which improved with the introduction of PCWP (*χ* ^2^ = 15.38; df = 1; *p* < 0.0001; Table 2, Tables S4–S6).

The same was observed in the Cpc‐PH cohort, where there was a significant association between PAC and PVR ($\beta_{1}$ = 1.24; 95% CI 0.95–1.52, *z* = 8.48, *p* < 0.0001; Table 2, Table S1), which was significantly modified by PCWP (*z* = 3.50; *p* < 0.0001).

Importantly, PCWP was not a significant modifier of the RC relationship in the pre‐capillary PH cohort ($\beta_{1}$ = 0.004; 95% CI −0.006 to 0.01; *z* = 0.849, *p* = 0.369; Table S2).

### 3.1 Modified pulmonary arterial compliance calculation (PAC_PCWP_ )

**FIGURE 4:**
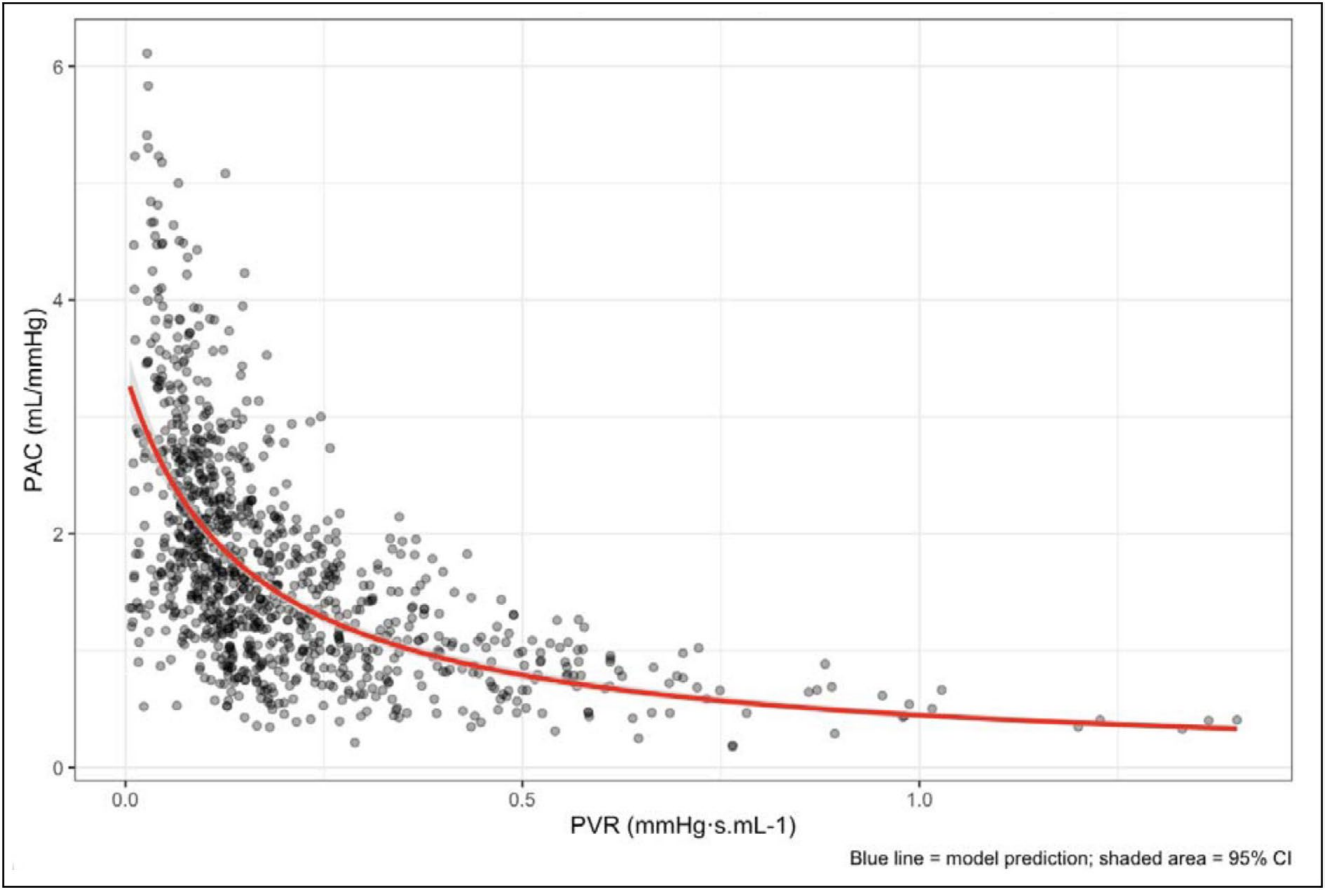
The PAC_PCWP_ to PVR (RC) relationship, PAC adjusted for PCWP. Gray bands represent confidence intervals.

The comparison between mean PAC_PCWP_ and PAC is shown in Table 3, The average PAC_PCWP_ was 1.74 ± 0.96 mL mmHg^−1^, which was significantly lower than the PAC, 2.98 ± 1.90 mL mmHg^−1^ (*t* = 18.53, *p* < 0.001). There was a significant association between PAC_PCWP_ and PVR ($\beta_{1}$ = 1.97; 95% CI 1.77–2.11, *z* = 22.01, *p* < 0.0001; $\mathrm{PVR}=\frac{1}{0.30+1.97\cdot {\mathrm{PAC}}_{\text{pcwp}}}$; Table 4; Figure 4). However, model comparison statistics showed a stronger relationship between PAC and PVR than PAC_PCWP_ and PVR (AIC −2201.4; BIC −2176.8; *R* ^2^ 0.968 and AIC −2069.8; BIC 2045.2; *R* ^2^ 0.862, respectively; PAC_PCWP_; Table S7).

**TABLE 3:** Average pulmonary arterial compliance, PAC and PAC_pcwp_ methods.

Cohort	PAC_pcwp_ (mL mmHg^−1^)	PAC (mL mmHg^−1^)	*p*‐value	Sample size
Entire cohort	1.74 ± 0.96	2.98 ± 1.90	<0.0001	1017
Ipc‐PH	1.80 ± 0.73	3.57 ± 1.88	<0.0001	215
PVR <3WU	2.11 ± 0.98	3.74 ± 1.96	<0.0001	624
Cpc‐PH	0.99 ± 0.44	1.81 ± 0.93	<0.0001	292
Pre‐Capillary PH	1.58 ± 0.66	2.34 ± 1.24	<0.0001	280

Abbreviations: Cpc‐PH, combined pre‐ and post‐capillary pulmonary hypertension; Ipc‐PH, isolated pre‐ and post‐capillary pulmonary hypertension; PAC, pulmonary arterial compliance, calculated using the empiric formula; PAC_pcwp_, pulmonary arterial compliance, calculated using modified empiric formula, with diastolic pressure gradient; PVR, pulmonary vascular resistance.

**TABLE 4:** Results of PVR and PAC_pcwp_ regression analyses.

Cohort	Intercept (β_0_)	Coefficient ($\beta_{1}$)	95% CI	*z*‐value	*p*‐value	Sample size
[PAC]*	0.13	1.48	1.39, l1.57	31.62	<0.0001	1017
PAC_pcwp_	0.30	1.97	1.71; 2.11	22.01	<0.0001	1017
No PH	0.23	1.62	1.24, 2.01	8.35	<0.0001	230
Ipc‐PH	0.43	1.86	1.04, 2.67	4.46	<0.0001	215
PVR <3WU	0.29	2.20	1.84, 2.56	11.94	<0.0001	624
Cpc‐PH	0.61	1.88	1.49, 2.28	9.33	<0.0001	292
Pre‐capillary PH	0.27	1.45	1.32, 1.59	20.76	<0.0001	280

Abbreviations: CI, confidence interval; Ipc‐PH, isolated post‐capillary pulmonary hypertension; PAC, PAC calculated using the empiric formula, stroke volume divided by pulmonary pulse pressure; PAC_pcwp_, pulmonary arterial compliance incorporating pulmonary capillary wedge pressure; PH, pulmonary hypertension; PVR, pulmonary vascular resistance.

* PAC model for comparison.

In Ipc‐PH there was a strong relationship between PAC_PCWP_ and PVR ($\beta_{1}$ = 1.86; 95% CI 1.04–2.67, *z* = 4.46, *p* < 0.0001; $\mathrm{PVR}=\frac{1}{0.43+1.86\cdot {\mathrm{PAC}}_{\text{pcwp}}}$; Table 4). Notably, in the Ipc‐PH cohort, the *R* ^2^ statistic improved from 0.725 for PAC model, to 0.919 for PAC_PCWP_. Model fit was improved in the cohorts with isolated post‐capillary PH, low PVR (<3 WU), and normal pulmonary pressures, using PAC_PCWP_, whereas in cohorts with higher resistive loads (the pre‐capillary PH and Cpc‐PH cohorts), PAC performed more strongly.

## 4 DISCUSSION

The inverse hyperbolic RC relationship between PVR and PAC is well‐established and provides a useful framework for quantifying global RV afterload. PCWP influences this relationship such that rising PCWP reduces PAC. This raises questions about (1) the influence of PCWP on PAC in PH subgroups, especially those with elevated PCWP and (2) the validity of using the empiric PAC formula, which does not include the PCWP, in post‐capillary PH cohorts (Dupont et al., 2012; MacKenzie Ross et al., 2013; Metkus et al., 2016; Pagnamenta et al., 2013; Pellegrini et al., 2014; Tedford et al., 2012). In this study, we used real‐world hemodynamic data to examine the effect of PCWP on the PAC and RC relationship in PH subgroups and recommend an alternative method for calculating PAC to integrate the PCWP into the PAC estimation in these cohorts.

Key findings in this study included (i) the RC relationship persists at lower PVR values, including in cohorts with post‐capillary PH; however, (ii) the RC relationship is modulated by or dependent on PCWP in subgroups with higher PCWP and lower PVR, highlighting a need to consider incorporating PCWP into assessments of RV afterload, particularly in Ipc‐PH.

These findings demonstrate the effect of elevated PCWP in specific PH sub‐groups, and are additive to those from previous studies, showing the effect of elevated PCWP on the RC relationship in general PH cohorts. Further, while the RC relationship has been well validated in cohorts with primary PH and elevated PVR (Lankhaar et al., 2006, 2008; Lim & Gustafsson, 2020; MacKenzie Ross et al., 2013; Naeije & Delcroix, 2014; Tedford, 2014; Tedford et al., 2012), data have been limited in left‐sided PH and in patients with lower PVR.

Our analysis showed that the RC relationship held across all groups except the Ipc‐PH cohort; however, the results identified PCWP as a significant modifier of this relationship, consistent with prior observations (Tedford et al., 2012). As hypothesized, inclusion of PCWP into calculation of PAC (PAC_PCWP_) resulted in the restoration of the RC relationship in the Ipc‐PH cohort. Significantly, PCWP influenced the RC relationship in all cohorts with a post‐capillary PH component (Ipc‐PH and Cpc‐PH), as well as in those with low PVR (<3.0 WU), with the greatest effect demonstrated in cohorts with lower PVR and Ipc‐PH.

Notably, the PCWP did not modulate the RC relationship in pre‐capillary PH. This is in line with the theory that at higher PVR, pulmonary compliance is saturated, and further increases in PCWP therefore have little further impact on the RC relationship (Tedford et al., 2012).

The derivation of the PAC_PCWP_ equation is based on the physiological interpretation of PAC as the change in volume per unit change in pressure. In the empiric formula (SV/PP), PP represents the difference between peak systolic pressure and diastolic pressure. The purpose is that it represents the gradient across the pulmonary circulation, and may therefore more precisely be represented by the difference in the SPAP and pulmonary outflow pressure. In the pulmonary circulation, the outflow pressure is best approximated by the left atrial pressure, measured as PCWP. In normal physiology or pre‐capillary PH, the DPAP closely reflects or exceeds the outflow pressure, as PCWP remains low, and the PP therefore approximates this gradient. However, in post‐capillary PH, such as Ipc‐PH, where PCWP is elevated, it can dominate the outflow pressure, significantly altering the DPG (DPG = DPAP − PCWP), and giving a lower PP than would be calculated by SPAP − DPAP.

Incorporating PCWP into the PAC calculation (PAC_PCWP_) accounts for the additional pressure load from the left atrium and adjusts for these hemodynamic changes. While PAC_PCWP_ lowers the calculated PAC value, it more accurately reflects the load on the RV in the setting of an elevated outflow or left atrial pressure. This is supported by both the restoration of the RC relationship in Ipc‐PH, as well as the improvement in model fit in this cohort. Alternative methods for incorporating the PCWP into the PAC equation were explored, and these results are included in the Supplement. All methods for incorporating the PCWP, including using the DPG, which can have negative values (SV/(SPAP − DPG)), as well as using DPG, with DPG = 0 where DPG has negative values, yielded a similar effect on the RC relationship, which was significant compared to the empiric PAC formula (PAC = SV/PP). The method used in the main analysis (SV/(PP + PCWP)) was therefore selected as it is the most intuitive, adding the PCWP component of pressure load.

Importantly, PCWP significantly modified the RC relationship in patients with normal pulmonary pressures, and low PVR (<3 WU), suggesting that even modest elevations in PCWP can reduce compliance in otherwise normal pulmonary vasculature. This supports the idea that elevated PCWP increases transluminal pressure, reducing pulmonary vascular capacitance and contributing to RV afterload, even below diagnostic thresholds for PH. Finally, in this analysis cases with borderline PH, defined as mPAP 20–25 mmHg with normal PVR and PCWP were included within the group with normal pulmonary pressures. This subgroup comprised only 22 patients, and their inclusion did not materially affect the results of the analysis.

## 5 LIMITATIONS

Although this study is strengthened by a large real‐world dataset and provides robust evidence of the influence of PCWP on PAC and the RC relationship in left‐sided PH, further studies incorporating prognostic outcomes are needed to validate the clinical utility of PAC_PCWP_ in diagnostic and therapeutic decision‐making.

## 6 CONCLUSION

This study extends the current understanding of the RC relationship by demonstrating its persistence at lower PVR, as well as its modulation by PCWP in PH subgroups with lower PVR. Incorporating the PCWP into assessments of global RV load may enhance diagnostic accuracy, especially in patients with left‐sided PH. Future studies should explore the prognostic and therapeutic implications of these observations to further inform the clinical relevance and interpretation of these findings.

## FUNDING INFORMATION

The authors have nothing to report.

## CONFLICT OF INTEREST STATEMENT

The authors declare no conflicts of interest.

## ETHICS STATEMENT

De‐identified data may be made available upon reasonable request to the corresponding author, subject to approval by the Ethics Committee and in accordance with applicable data‐sharing agreements.

## Supporting information


Data S1.

## Data Availability

The data underlying this study contain sensitive patient‐level information and cannot be made publicly available due to institutional and ethical restrictions.
