# Supplementary material for: The resistance‐compliance relationship at low pulmonary resistance: Integrating pulmonary wedge pressure into right ventricular load assessment
Source: Physiol Rep. 2026 Mar 8;14(5):e70811. doi: 10.14814/phy2.70811 (PMC12967464; doi:10.14814/phy2.70811)
Supplement: Supplementary file 1 — Data S1. [file PHY2-14-e70811-s001.docx]

**Supplement**

*Alternative methods for incorporating the PCWP into the PAC equation*

In addition to the primary PCWP-based formulation, several alternative PAC calculations were evaluated as sensitivity analyses, which is shown in Figure 1. These included: (1) empiric PAC definition based on stroke volume divided by pulmonary pulse pressure (red); (2) incorporation of the DPG into the denominator (yellow); (3) substitution of the absolute value of DPG, i.e. the pulmonary pulse pressure with the addition of PCWP, as used in the main manuscript (green); (4) truncation of negative DPG values to zero (blue); and (5) use of the higher of DPAP or PCWP as the downstream pressure term (pink). This last method is not favoured, as it departs further from the definition of PAC, neglecting the pulse pressure. Each PAC definition was calculated on a per-patient basis and examined in relation to PVR, as shown in Figure S1.


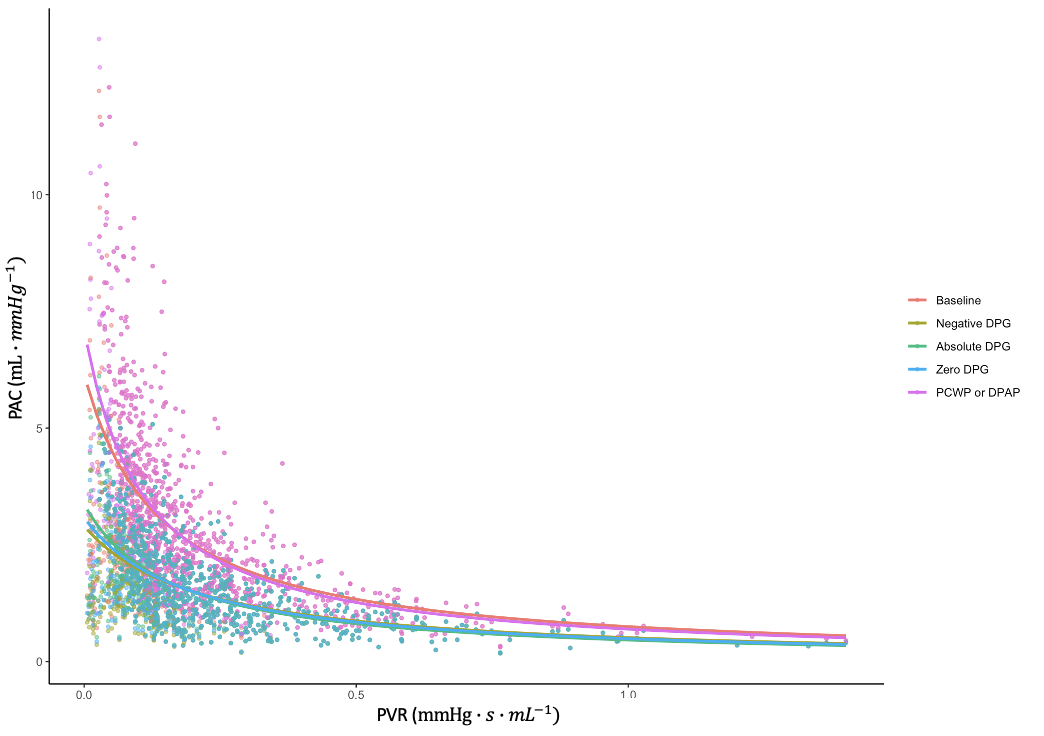


Figure S1: Relationship between PAC and PVR across alternative PAC calculation methods. Each point represents an individual case. Solid lines depict inverse hyperbolic fits illustrating the RC relationship for each PAC formulation. DPG-based approaches (negative DPG, absolute DPG, and DPG truncated at zero) show closely overlapping RC relationships, whereas formulations incorporating PCWP or DPAP demonstrate a distinct shift in the PAC-PVR relationship.

Differences in absolute PAC values across calculation methods were assessed using a Friedman test for repeated measures, to assess whether PAC values differed systematically across calculation methods within the same cases. PAC values differed significantly across methods (χ² = 3829.5, df = 4, p < 0.001). Pairwise Wilcoxon signed-rank tests with Holm correction to control the error rate, demonstrated statistically significant differences between all PAC definitions. These findings reflect systematic algebraic differences introduced by the alternative formulations, however, paired differences were small relative to the overall range of PAC values.

To evaluate whether the method used to calculate PAC influenced the relationship between PAC and PVR, generalised linear mixed models with Gamma distribution and inverse link functions were fitted, consistent with the established inverse hyperbolic form of the RC relationship (likelihood ratio test χ² = 779.1, df = 8, p < 0.001), indicating that the RC relationship differed depending on the PAC formulation. These differences were driven primarily by formulations incorporating either PCWP or DPAP (method 5 above), whereas alternative DPG-based approaches yielded similar PAC-PVR relationships.

**Table S1:** Model comparison statistics*

| **Model** | **AIC** | **BIC** | **PseudoR^2^** | **ANOVA** (χ^2^, df, p-value)** |
| --- | --- | --- | --- | --- |
| Entire cohort (n = 1017) | | | | |
| 1 | -2185.1 | -2165.4 | 0.968 |  |
| 2 | -2331.9 | -2297.4 | 0.985 | χ^2^ = 152.71; df = 3; p < 0.0001 |
| Normal/borderline PH (n = 230) | | | | |
| 3 | -907.8 | -894.0 | 0.974 |  |
| 4 | -929.4 | -905.3 | 0.984 | χ^2^ = 27.62; df = 3; p < 0.0001 |
| Ipc-PH (n = 215) | | | | |
| 5 | -787.8 | -767.6 | 0.725 |  |
| 6 | -790.65 | -767.05 | 0.881 | χ^2^ = 4.81; df = 3; p = 0.028 |
| Low PVR cohort; PVR <3.0 WU (n = 624) | | | | |
| 7 | -2147.6 | -2129.8 | 0.951 |  |
| 8 | -2158.8 | -2127.7 | 0.963 | χ^2^ = 17.15; df = 3; p < 0.0001 |
| Pre-capillary PH (n = 280) | | | | |
| 9 | -562.1 | -547.5 | 0.966 |  |
| 10 | -582.83 | -557.39 | 0.972 | χ^2^ = 26.76; df = 3; p < 0.0001 |
| Cpc-PH (n = 292) | | | | |
| 11 | -612.1 | -597.4 | 0.367 |  |
| 12 | -620.7 | -594.9 | 0.489 | χ^2^ = 14.57; df = 3; p = 0.002 |

*Results can only be compared between model pairs; ** likelihood ratio for model comparison

AIC: Akaike information criterion; BIC: Bayesian information criterion**;** Cpc-PH: combined pre- and post-capillary pulmonary hypertension; Ipc-PH: Isolated post-capillary pulmonary hypertension; PAC: pulmonary arterial compliance; PH: pulmonary hypertension; PVR: pulmonary vascular resistance; WU: Wood units

**Table S2:** Simple regression analyses for PAC (mL.mmHg^-1^) and PCWP (mmHg)

| **Cohort** | **Intercept (**$\boldsymbol{\beta}_{\boldsymbol{0}}$**)** | **Coefficient (**$\boldsymbol{\beta}_{\boldsymbol{1}}$**)** | **Confidence interval 95%** | **Z score** | **P value** | **Sample size** |
| --- | --- | --- | --- | --- | --- | --- |
| Entire cohort | 0.17 | 0.01 | 0.008; 0.012 | 11.55 | <0.0001 | 1017 |
| Normal/borderline PH | 0.21 | 0.0005 | -0.003; 0.004 | 0.24 | 0.814 | 230 |
| Ipc-PH | 0.03 | 0.01 | 0.008; 0.015 | 6.35 | < 0.0001 | 215 |
| PVR <3.0 WU | 013 | 0.009 | 0.007; 0.01 | 12.33 | <0.0001 | 624 |
| Pre-capillary PH | 0.38 | 0.004 | -0.006; 0.01 | 0.85 | 0.396 | 280 |
| Cpc- PH | 0.29 | 0.01 | 0.007; 0.02 | 4.68 | <0.0001 | 280 |

Cpc-PH: combined pre- and post-capillary pulmonary hypertension; I-pc PH: Isolated post-capillary pulmonary hypertension; PAC: pulmonary arterial compliance; PH: pulmonary hypertension; PVR: pulmonary vascular resistance; WU: Wood units

**Table S3:** Simple regression analyses for PVR (mmHg/L.min^-1^) and PCWP (mmHg)

| **Cohort** | **Intercept**  **(**$\boldsymbol{\beta}_{\boldsymbol{0}}$**)** | **Coefficient (**$\boldsymbol{\beta}_{\boldsymbol{1}}$**)** | **Confidence interval 95%** | **Z score** | **P value** | **Sample size** |
| --- | --- | --- | --- | --- | --- | --- |
| Entire cohort | 4.67 | 0.02 | -0.01; 0.05 | 1.13 | 0.260 | 1017 |
| Normal/borderline PH | 6.00 | 0.56 | 0.33; 0.78 | 4.84 | <0.0001 | 230 |
| Ipc-PH | 10.76 | 0.08 | -0.07; 0.24 | 1.03 | 0.303 | 215 |
| PVR <3.0 WU | 10.70 | -0.04 | -0.09; 0.01 | -1.56 | 0.119 | 624 |
| Pre-capillary PH | 3.06 | 0.001 | -0.08; 0.08 | 0.03 | 0.976 | 280 |
| Cpc- PH | 3.12 | 0.02 | -0.02; 0.05 | 0.947 | 0.344 | 280 |

Cpc-PH: combined pre- and post-capillary pulmonary hypertension; I-pc PH: Isolated post-capillary pulmonary hypertension; PAC: pulmonary arterial compliance; PH: pulmonary hypertension; PVR: pulmonary vascular resistance; WU: Wood units

Multivariable modelling was performed to examine the effect of PCWP as a covariate in the following groups.

1. Entire cohort
2. (1) adjusted for PCWP, age and BSA
3. Normal/borderline PH
4. (3) adjusted for PCWP, age and BSA
5. Ipc-PH
6. (5) adjusted for PCWP, age and BSA
7. PVR <3WU
8. (7) adjusted for PCWP, age and BSA
9. Pre-capillary PH
10. (9) adjusted for PCWP, age and BSA
11. Combined pre-and post-capillary PH
12. (11) adjusted for PCWP, age and BSA

**Table S4:** coefficients for PCWP in adjusted models for PAC and PVR (adjusted for age, BSA and PCWP)

| **Model** | **Coefficient** | **95%CI** | **Z Score** | **p-value** | **Sample size** |
| --- | --- | --- | --- | --- | --- |
| 2 (entire cohort) | 0.10 | 0.08; 0.12 | 10.86 | <0.0001 | 1017 |
| 4 (normal/borderline PH) | 0.58 | 0.37; 0.79 | 5.47 | <0.0001 | 230 |
| 6 (Ipc-PH) | 0.19 | 0.02; 0.36 | 2.16 | 0.031 | 215 |
| 8 (PVR <3WU) | 0.11 | 0.06; 0.17 | 3.91 | <0.0001 | 624 |
| 10 (pre-capillary PH) | 0.05 | 0.01; 0.08 | 2.55 | 0.011 | 280 |
| 12 (Cpc-PH) | 0.005 | 0.02; 0.07 | 3.50 | <0.0001 | 280 |

**Table S5:** coefficients for age in adjusted models for PAC and PVR (adjusted for age, BSA and PCWP)

| **Model** | **Coefficient** | **95%CI** | **Z Score** | **p-value** | **Sample size** |
| --- | --- | --- | --- | --- | --- |
| 2 (entire cohort) | 0.018 | 0.012; 0.024 | 5.90 | <0.001 | 1017 |
| 4 (normal/borderline PH) | 0.01 | -0.02; 0.05 | 0.55 | 0.581 | 230 |
| 6 (Ipc-PH) | 0.01 | -0.05; 0.07 | 0.31 | 0.758 | 215 |
| 8 (PVR <3WU) | 0.02 | -0.009; 0.04 | 1.25 | 0.211 | 624 |
| 10 (pre-capillary PH) | 0.01 | 0.007; 0.18 | 4.19 | <0.001 | 280 |
| 12 (Cpc-PH) | 0.01 | 0.003; 0.03 | 2.39 | 0.017 | 280 |

**Table S6:** coefficients for BSA in adjusted models for PAC and PVR (adjusted for age, BSA and PCWP)

| **Model** | **Coefficient** | **95% CI** | **Z Score** | **p-value** | **Sample size** |
| --- | --- | --- | --- | --- | --- |
| 2 (entire cohort) | -0.72 | -1.23; -0.21 | -2.75 | 0.006 | 1017 |
| 4 (normal/borderline PH) | -0.31 | -3.02; 2.4 | -0.22 | 0.825 | 230 |
| 6 (Ipc-PH) | -2.61 | -6.33; 1.10 | -1.38 | 0.168 | 215 |
| 8 (PVR <3WU) | -1.92 | -3.43; - 0.42 | -2.51 | 0.010 | 624 |
| 10 (pre-capillary PH) | -0.45 | -0.92; 0.02 | -1.86 | 0.062 | 280 |
| 12 (Cpc-PH) | 0.11 | -0.61; 0.83 | 0.29 | 0.771 | 280 |

**Table S****7:** Model comparison statistics for sub-cohort analyses, PAC_pcwp_ and PAC***

| **Method** | **AIC** | **BIC** | **Pseudo-R2 statistic** | **Sample size** |
| --- | --- | --- | --- | --- |
| Entire Cohort | | | | |
| PAC | -2185.1 | -2165.4 | 0.968 | 1017 |
| PAC_pcwp_ | -2065.6 | -2045.9 | 0.862 | 1017 |
| No PH | | | | |
| PAC | -907.8 | -894.0 | 0.974 | 230 |
| PAC_pcwp_ | -921.2 | -907.5 | 0.979 | 230 |
| Ipc-PH | | | | |
| PAC | -791.05 | -777.13 | 0.725 | 215 |
| PAC_pcwp_ | -800.61 | -787.13 | 0.919 | 215 |
| PVR <3WU | | | | |
| PAC | -2147.6 | -2129.8 | 0.951 | 624 |
| PAC_pcwp_ | -2165.1 | -2147.3 | 0.957 | 624 |
| Cpc-PH | | | | |
| PAC | -612.1 | -597.4 | 0.367 | 292 |
| PAC_pcwp_ | -598.8 | -584.10 | 0.274 | 292 |
| Pre-Capillary PH | | | | |
| PAC | -562.0 | -547.5 | 0.966 | 280 |
| PAC_pcwp_ | -538.4 | -523.8 | 0.961 | 280 |

*Results can only be compared between model pairs; AIC: Akaike information criterion; BIC: Bayesian information criterion; Ipc-PH: isolated post-capillary pulmonary hypertension; PAC_pcwp_: pulmonary arterial compliance calculated incorporating the pulmonary capillary wedge pressure; PAC: PAC calculated using the empiric formula; PVR: pulmonary vascular resistance
